# A novel ophthalmic viscosurgical device-free phakic intraocular lens implantation makes myopic surgery safer

**DOI:** 10.1186/s40662-020-00185-4

**Published:** 2020-04-07

**Authors:** An-Peng Pan, Li-Jin Wen, Xu Shao, Kai-Jing Zhou, Qin-Mei Wang, Jia Qu, A-Yong Yu

**Affiliations:** The Eye Hospital of Wenzhou Medical University, 270 Xueyuan West Road, Wenzhou, 325000 Zhejiang P. R. China

**Keywords:** Phakic intraocular lens implantation, Ophthalmic viscosurgical device-free, Intraocular pressure, Complications

## Abstract

**Purpose:**

To assess the efficacy and safety of a novel ophthalmic viscosurgical device-free (OVD-free) method for posterior chamber phakic intraocular lens (PIOL) implantation in myopic eyes.

**Methods:**

In this retrospective cohort study, the medical records of myopic eyes that underwent PIOL (Implantable Collamer Lens, ICL) implantation for myopia correction at the Eye Hospital of Wenzhou Medical University between May 2015 and March 2017 were reviewed. A total of 49 eyes with complete data that met follow up requirements (2 h, 1 day, 1 week, 3 months postoperatively) were recruited. Based on the surgical techniques used, the eyes were divided into the OVD-free method group and the standard method group. The clinical data, including intraocular pressure (IOP), corrected distance visual acuity (CDVA) and spherical equivalent (SE), at each follow-up were collected for comparison. Endothelial cell loss and complications were also investigated.

**Results:**

Twenty-one eyes received the standard method, and 28 eyes received the OVD-free method. A rise in IOP > 22 mmHg at 2 h was noted in 14 eyes (66.7%) in the standard group and none (0%) in the OVD-free group (*p* < 0.001). The rise in IOP from baseline was significantly higher at 2 h in the standard group (10.5 ± 5.2 mmHg vs. 2.2 ± 3.3 mmHg, difference: 8.3, 95% CI 5.8 to 10.8; *p* < 0.001). There was a significant difference in the time course of LogMAR CDVA changes between the two groups (*p* = 0.047). The LogMAR CDVA was significantly better in the OVD-free method group compared to the standard group at 1 day (− 0.076, 95% CI − 0.134 to − 0.018; *p* = 0.012), 1 week (− 0.071, 95% CI − 0.135 to − 0.007; *p* = 0.03), but not at 3 months (− 0.046, 95% CI − 0.107 to 0.015; *p* = 0.134). There was no significant difference in the time course of SE changes between the two groups (*p* = 0.471; *p* = 0.705). In the OVD-free group, mean endothelial cell loss was 4.6% at 3 months (2522 ± 281 vs. 2407 ± 226 cells/mm^2^, difference: -115, 95% CI − 295 to 65; *p* = 0.187). No complications were reported in both groups except for the early IOP elevation in the standard group during the observation period.

**Conclusions:**

The OVD-free method is safe and efficient for ICL implantation. It can be a safer method of ICL implantation compared to the standard method in that it completely eliminates ophthalmic viscoelastic devices-related complications without causing additional complications.

## Background

Myopia is one of the most common disorders of the eye in the world, and its prevalence is continuing to increase [1–4]. The estimated number of people with myopia was 1406 million worldwide in 2000 and is predicted to reach about 4760 million by 2050 [5]. Posterior chamber phakic intraocular lens (PIOL) implantation has been reported to be a safe and effective surgical option for myopia correction [6–10]. Higher reversibility and less loss of best spectacle-corrected visual acuity have made this procedure more favorable than laser refractive surgery in patients with moderate to high myopia [11–14]. For example, Implantable Collamer Lens (ICL; STAAR Surgical Co, Monrovia, California) is one of the commonly used PIOLs. Over 700,000 ICLs had been implanted to date, and over 120,000 surgeries are performed in over 60 countries annually. With the increasing performance of ICL implantation, the potential complications such as early (within 24 h postoperatively) acute intraocular pressure (IOP) elevation should be minimized [15, 16]. Early acute IOP elevation was reported to be relatively frequent due to the retained ophthalmic viscosurgical devices (OVDs) [15, 17–19]. On the other hand, the intraoperative use of OVDs may increase the cost of consumable material and overall operation time due to the extra steps of injection and, late removal of OVDs [20]. The prolonged surgery and extra aggressive steps of irrigating the OVDs may increase the chance of inadvertent ICL-crystalline lens contact and will lead to early lens opacification after surgery [15, 21, 22].

Injection of OVDs seems to be crucial and necessary to maintain the stability of the anterior chamber during standard surgery procedure. Therefore, to avoid the usage of OVDs which can lead to OVD-related complications, we invented an ophthalmic viscosurgical device-free (OVD-free) method with unique surgical instruments for PIOL implantation. The anterior chamber can then be well infused and maintained without the use of OVDs. In this study, we demonstrate that this patented OVD-free method (IP Australia 2,016,234,979, http://pericles.ipaustralia.gov.au/ols/auspat/applicationDetails.do?applicationNo=2016234979), which uses a continuous infusion with balanced salt solution (BSS) through a side-port produces no OVD-related complications and can also simplify the surgical procedure. The purpose of this study was to investigate the efficacy and safety of the OVD-free PIOL implantation in myopic eyes by comparing the complications and clinical outcomes of the OVD-free method with the standard method.

## Methods

### Study design and participants

The medical records of myopic eyes that underwent ICL (V4c) implantation for myopia correction at the Eye Hospital of Wenzhou Medical University between May 2015 and March 2017 were reviewed. A total of 49 eyes with complete data that met follow up requirements were recruited. The study was designed and conducted by the investigators at the Eye Hospital of Wenzhou Medical University who also collected, managed, and analyzed the data. Ethics approval was obtained from the institutional review board of the Eye Hospital of Wenzhou Medical University and the study was carried out in accordance with the tenets of Declaration of Helsinki. Signed informed consent concerning the risks of surgery was obtained from each patient.

The preoperative systemic and ocular history, refractive status, slit-lamp biomicroscopy, endothelial cell density, biometry, pachymetry, IOP, topographic keratometry, and ICL parameters were carefully reviewed. The clinical data at each postoperative follow-up visit were collected, including the noncontact tonometry (TX-F; Canon, Tokyo, Japan) at 2 h, 1 day, 1 week, 3 months postoperatively, corrected distance visual acuity (CDVA) and spherical equivalent (SE) at 1 day, 1 week, 3 months postoperatively, endothelial cell density (ECD) at 3 months postoperatively, and slit-lamp examinations at all time points. A complete analysis of adverse complications was performed.

### Intraocular lens

The ICL V4c model was used in all 49 patients. A 0.36 mm hole in the center of the ICL optic is designed to improve the circulation of the aqueous humor and eliminated the need for a preoperative laser iridotomy or intraoperative iridectomy [23, 24]. The postoperative targeted refraction was emmetropia in all eyes. Power calculation for the ICL was performed using the software provided by the manufacturer with a modified vertex formula. The ICL diameter was determined following the manufacturer’s recommendations based on the horizontal white-to-white distance measured by a caliper, and the anterior chamber depth measured by Scheimpflug imaging system (Pentacam HR; Oculus GmbH, Wetzlar, Germany).

### Surgical technique

All surgeries were performed by the same surgeon (A.Y.). The surgeries were performed under pupil dilation with 0.5% tropicamide and 0.5% phenylephrine hydrochloride eye drops (Zhuobian, Sinqi, Shenyang, China). In the standard method group, the brief procedures of surgery were as follows: After topical anesthesia, a 3.0 mm temporal clear corneal main incision and one side-port (1.0 mm in size) were created. The anterior chamber was filled with an OVD (1.7% sodium hyaluronate, Bausch & Lomb, Zhengda Freedom Group, Shandong, China). The ICL was inserted through the main incision with the use of an injector cartridge (STAAR Surgical, Switzerland), and was placed in the posterior chamber by gently tucking the footplates beneath the iris. The OVD was manually irrigated out of the anterior chamber with BSS. No preoperative or intraoperative peripheral iridectomy was performed in any case.

**Fig. 1:**
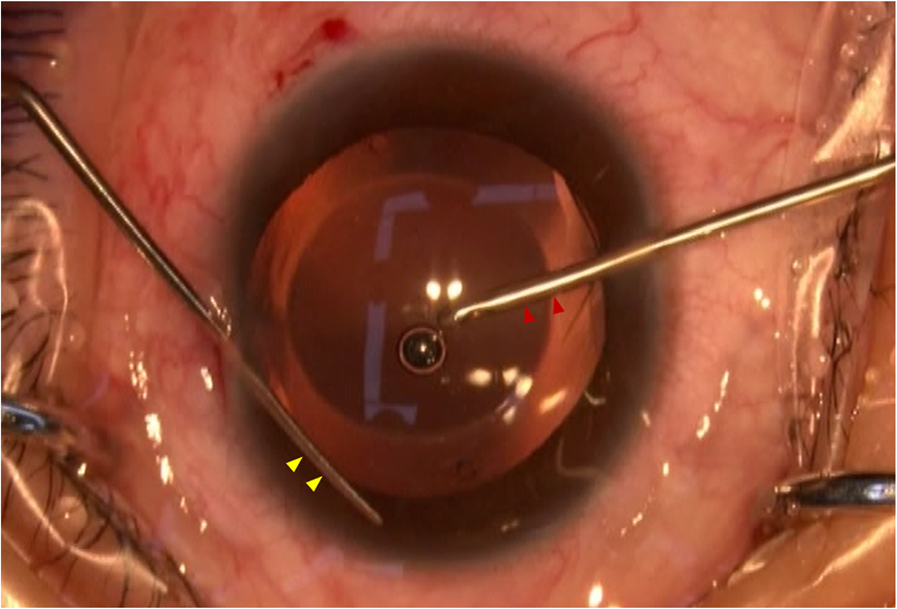
An intraoperative image of Ophthalmic Viscosurgical Device-free method of phakic intraocular lens (PIOL) implantation. The red triangles show the patent irrigator, the yellow triangles indicate the patent manipulator, the anterior chamber was well maintained during the whole surgical procedure

An intraoperative image of the OVD-free method is shown in Fig. 1. In the OVD-free method group, the surgical procedure was identical to the standard method except: 1. Two side-ports were created, one (0.5 mm in size) was used for continuous infusion with BSS by the patent irrigator (Fig. 1, red triangle) to fill and maintain the anterior chamber, and the another (0.3 mm in size) was used for tucking the footplates by the patent manipulator (Fig. 1, yellow triangle); 2. No OVD was used, and thus completely eliminating the need for irrigation of the OVD at the end of the surgery. The differences between the two methods are listed in Table 1.

**Table 1:** Comparison of the two methods of phakic intraocular lens (PIOL) implantation

	OVD-free PIOL implantation	Standard PIOL implantation
Number of side port(s)	2	1, or none
OVD usage	No	Yes
OVD removal	No	Yes
Unfolding of the PIOL	Quick	Slow
Consumption of BSS	Less	More
Duration of surgery	Short	Long

*OVD* = Ophthalmic Viscosurgical Device

The operation time was calculated for each surgery (the time calculated from making the first corneal incision to hydrating the incision at the end). After surgery, antibiotic eye drops (0.5% Levofloxacin Eye Drops, Santen Pharmaceutical Co. Ltd., Japan) and anti-inflammatory corticosteroid (0.5% loteprednol etabonate ophthalmic suspension, Bausch & Lomb, Florida, USA) were administered topically 4 times daily for 1 week and then gradually tapered, nonsteroidal anti-inflammatory eye drops (0.1% pranoprofen, Senju Pharmaceutical Co., Ltd. Fukusaki Plant, Japan) were administered topically 4 times daily for 2 weeks.

### Management of early acute IOP elevation

Rise in IOP > 22 mmHg at 2 h after surgery was defined as early acute IOP elevation. Once early acute IOP elevation was noted, timely management was required: 1. A slit-lamp examination was performed to evaluate the vault and also identify any sign of serious etiology such as pupillary block or malignant glaucoma. 2. Mild IOP elevation (< 25 mmHg) required close observation; moderate IOP elevation (≥25 mmHg, ≤30 mmHg) required topical antiglaucoma medications; severe IOP elevation (> 30 mmHg) required anterior chamber drainage through the side-port with or without topical antiglaucoma medications; surgical intervention of anterior chamber irrigation or even ICL exchange were reserved for uncontrolled IOP elevation. 3. The eye was re-evaluated and treated appropriately every hour and treated appropriately until the IOP returned to the normal range.

### Statistical analysis

Data analysis was performed using SPSS 25.0 (IBM Corp., New York, NY, USA). Visual acuity data were converted to LogMAR values for statistical analysis. All continuous variables were expressed as the mean ± standard deviation, and categorical variables were summarized as percentages. The Shapiro-Wilk test was used for testing normality. Independent-samples t test and Wilcoxon sum rank test were used for normally and nonnormally distributed data between two groups, respectively. Two-factor analysis of variance (ANOVA) with repeated measures was used for the analysis of the time course of changes intra- and inter-groups. Bonferroni correction was used for further comparisons between each two-time point intra-group and at each time point between two groups. The Chi-squared test was used to compare the proportions (IOP elevation, gender) between two groups. A *p*-value of less than 0.05 was considered significant.

## Results

Twenty-one eyes (twelve patients) received the standard method, and 28 eyes (16 patients) received the OVD-free method. Table 2 shows the preoperative demographic data of the patients and the PIOL characteristics.

**Table 2:** Preoperative patient demographics in eyes undergoing either method of phakic intraocular Lens (PIOL) implantation

Demographic	OVD-free method	Standard method	*P* value
Age (SD), year	28.50 (3.97)	26.81 (6.04)	0.243
Sex (%, female)	50.0%	85.7%	0.009
SE (SD), D	−13.09 (3.26)	− 13.49 (3.27)	0.676
AL (SD), mm	28.53 (1.62)	28.15 (1.73)	0.439
UDVA (IQR), logMAR	1.22 (1.02–1.40)	1.30 (1.16–1.40)	0.342
CDVA (IQR), logMAR	0.10 (0–0.15)	0.15 (0.05–0.30)	0.103
ACD (IQR), mm	3.59 (3.46–3.81)	3.73 (3.64–3.78)	0.084
CCT (IQR), μm	538.5 (518.0–564.8)	523.0 (492.5–560.0)	0.196
ECD (IQR), cells/mm^2^	2620.6 (2431.9–2983.9)	2620.6 (2490.7–2963.0)	0.664
IOP (IQR), mmHg	14.45 (12.65–15.95)	15.10(13.80–17.40)	0.122
PIOL Power (SD), D	−13.88 (2.78)	−14.93(2.18)	0.158

*OVD* = Ophthalmic Viscosurgical Device; *SE* = Spherical equivalent; *AL* = Axial length; *UDVA* = Uncorrected distance visual acuity; *CDVA* = Corrected distance visual acuity; *ACD* = Anterior chamber depth; *CCT* = Center corneal thickness; *ECD* = Endothelial cell density; *IOP* = Intraocular pressure; *SD* = Standard deviation; *IQR* = Interquartile range

### Postoperative intraocular pressure

Rise in IOP > 22 mmHg at 2 h was noted in fourteen of twenty-one eyes (66.7%) in the standard method group and none of the 28 eyes (0%) in the OVD-free method group (*p* < 0.001).

**Fig. 2:**
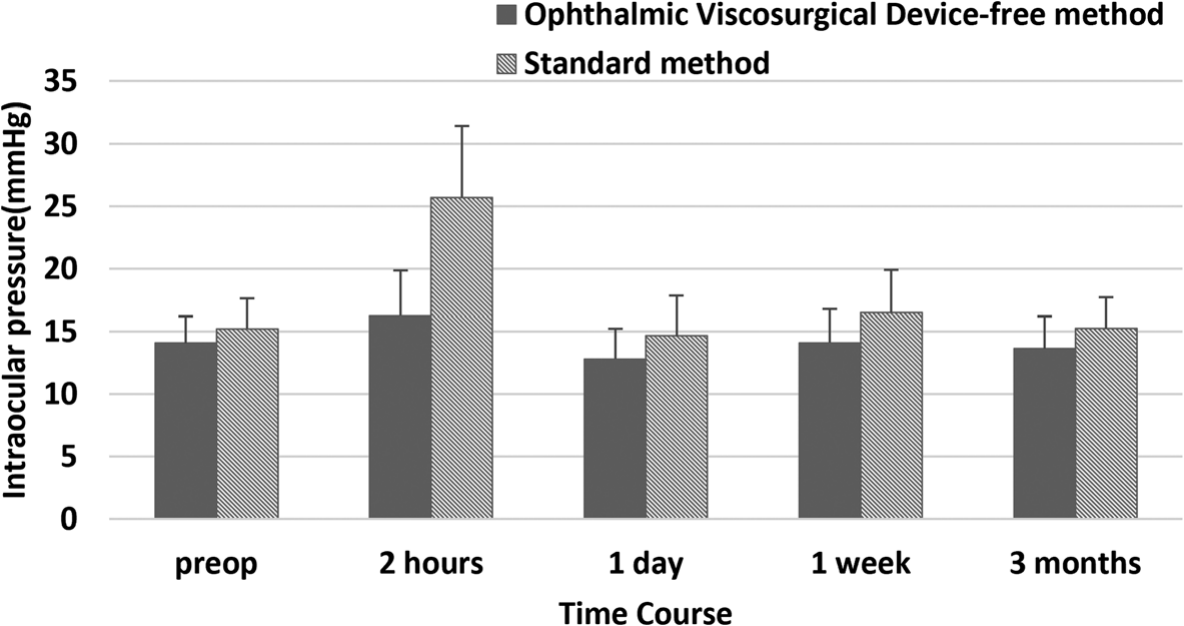
Time course of intraocular pressure changes in eyes undergoing the standard method of phakic intraocular lens (PIOL) implantation and Ophthalmic Viscosurgical Device-free method of PIOL implantation. Error bars represent standard error of mean

**Fig. 3:**
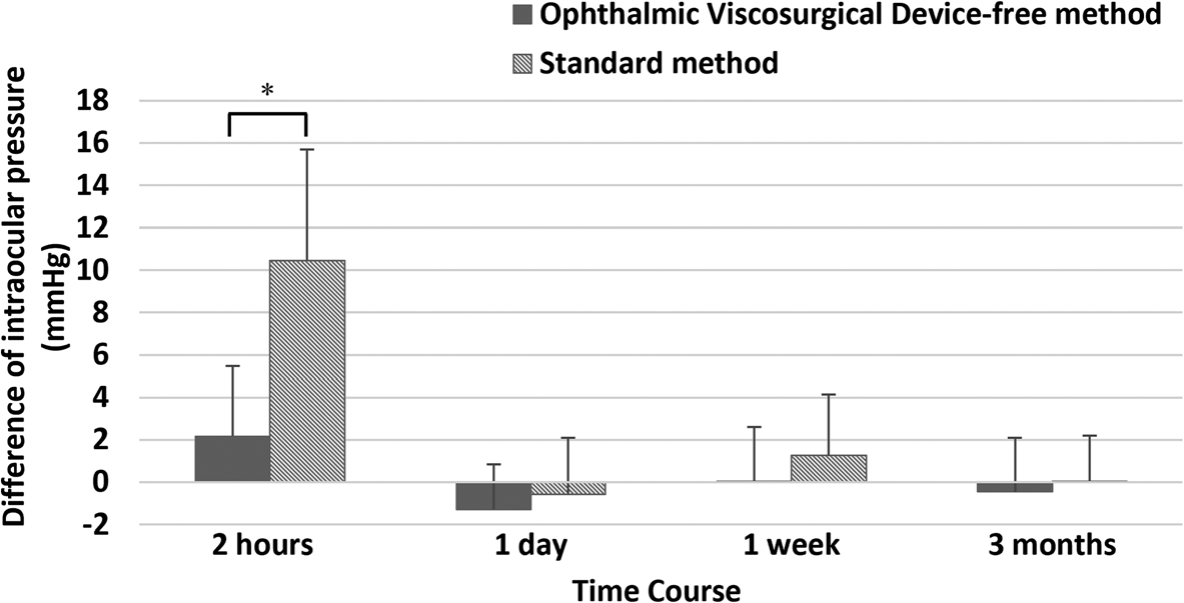
The difference of intraocular pressure between baseline (preoperation) and each time point in eyes undergoing the standard method of phakic intraocular lens (PIOL) implantation and Ophthalmic Viscosurgical Device-free method of PIOL implantation. Error bars represent standard error of mean. The asterisk represents statistically significant differences between the two groups (8.3, 95% CI 5.8 to 10.8; *p* < 0.001)

The time course of IOP changes was significantly different between the two groups (*p* < 0.001) (Fig. 2). At 2 h postoperatively, the IOP increased to 16.3 ± 3.6 mmHg in the OVD-free method group (2.2, 95% CI − 0.19 to 4.5; *p* = 0.094), and significantly increased to 25.7 ± 5.7 mmHg in standard method group (10.5, 95% CI 7.8 to 13.2; *p* < 0.001) (OVD-free method group vs standard method group: -9.4, 95% CI − 12.1 to − 6.8; *p* < 0.001). The difference of IOP between baseline (preoperation) and each time point was calculated for two groups (Fig. 3). At 2 h, 1 day, 1 week, and 3 months, the differences in the OVD-free method group were 2.2 ± 3.3 mmHg, − 1.3 ± 2.1 mmHg, 0 ± 2.6 mmHg, and − 0.5 ± 2.5 mmHg, respectively; and in standard method group, 10.5 ± 5.2 mmHg, − 0.6 ± 2.7 mmHg, 1.3 ± 2.9 mmHg, and 0 ± 2.2 mmHg, respectively. In inter-group comparisons, the difference of IOP from baseline level was significantly higher in the standard group compared to the OVD-free method group at 2 h (8.3, 95% CI 5.8 to 10.8; *p* < 0.001), but not at 1 day (0.7, 95% CI − 0.7 to 2.1; *p* = 0.303), 1 week (1.2, 95% CI − 0.3 to 2.8; *p* = 0.122) or 3 months (0.5, 95% CI − 0.9 to 1.9; *p* = 0.498).

### Visual acuity and refractive outcomes

At 1 day, 1 week and 3 months, the mean or median LogMAR CDVA in the OVD-free method group were 0.00 (IQR, 0.00 to 0.03), 0.00 (IQR, − 0.06 to 0.00), 0.00 (IQR, 0.00 to 0.00), respectively; and in the standard method group, 0.05 (IQR, 0.00 to 0.22), 0.08 (SD, 0.10), 0.00 (IQR, 0.00 to 0.13), respectively. The safety indices (mean postoperative CDVA/mean preoperative CDVA) in the OVD-free method group were 1.15, 1.21, 1.18, respectively; and in the standard method group, 1.13, 1.19, 1.23, respectively. There was a significant difference in the time course of LogMAR CDVA changes between the two groups (*p* = 0.047). In inter-group comparisons, the LogMAR CDVA was significantly better in the OVD-free method group compared with the standard group at 1 day (− 0.076, 95% CI − 0.134 to − 0.018; *p* = 0.012), 1 week (− 0.071, 95% CI − 0.135 to − 0.007; *p* = 0.03), but not at 3 months (− 0.046, 95% CI − 0.107 to 0.015; *p* = 0.134).

**Fig. 4:**
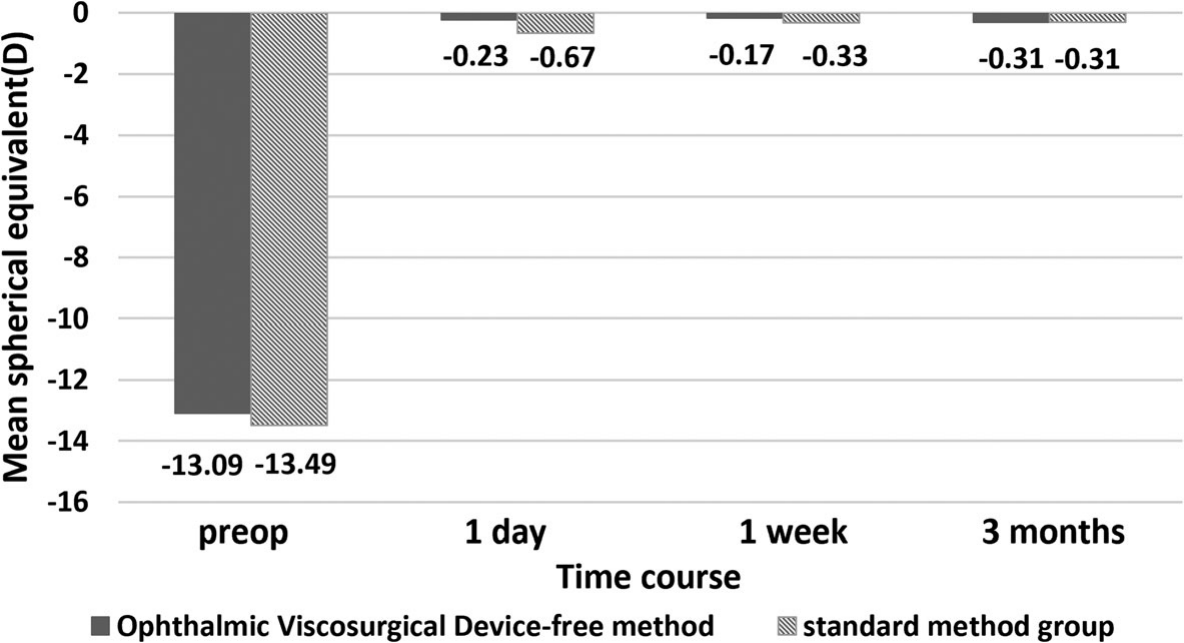
Time course of spherical equivalent changes in eyes undergoing the standard method of phakic intraocular lens (PIOL) implantation and Ophthalmic Viscosurgical Device-free method of PIOL implantation

Fig. 4 shows the changes of the mean SE over time in both groups. There was no significant difference in the time course of SE changes between the two groups (*p* = 0.524). At 3 months postoperatively, the mean SE was − 0.31 ± 0.73 D in the OVD-free method group, and − 0.30 ± 1.03 D in the standard method group. The improvement of SE from baseline was statistically significant in both groups (OVD-free method group: 12.78, 95% CI 11.35 to 14.22; standard method group: 13.19, 95% CI 11.53 to 14.85; all *p* < 0.001).

### Endothelial cell density

Only endothelial cell density measurements in the OVD-free method group at 3 months are available in this study. Endothelial cell density decreased from 2522 ± 281 cells/mm^2^ preoperatively to 2407 ± 226 cells/mm^2^ postoperatively (twelve eyes, paired t test, − 115, 95% CI − 295 to 65; *p* = 0.187). Mean endothelial cell loss was 4.6% at 3 months postoperatively.

### Operation time

In the OVD-free method group, the distribution of the operation time was three eyes (10.7%) for 1 min, 14 eyes (50.0%) for 2 min and eleven eyes (39.3%) for 3 min; In the standard method group, the distribution of the operation time was one eye (4.8%) for 2 min, five eyes (23.8%) for 3 min, ten eyes (47.6%) for 4 min, four eyes (19.0%) for 5 min and one eye (4.8%) for 6 min. The operation time was significantly shorter in the OVD-free group (*p* < 0.001).

### Complications

All surgical procedures were uneventful with no pupillary block, cataract formation or pigment dispersion syndrome observed during the 3-month observation period. The vaults were between 1/2 center corneal thickness and 2 center corneal thickness in all eyes checked under slit-lamp examinations. Early acute IOP elevation (> 22 mmHg) at 2 h was noted in 66.7% of eyes in the standard method group due to retained OVDs, and was lowered down by appropriate management, including invasive intervention such as anterior chamber drainage through the side-port as listed in Table 3. Early acute IOP elevation did not occur in the OVD-free method group.

**Table 3:** Management of early acute intraocular pressure elevation in 14 eyes following the standard method of phakic intraocular lens (PIOL) implantation

Management	Number of eyes
Without treatment, No. (%)	3 (21)
AC drainage only, No. (%)	6 (43)
TAM combined with AC drainage, No. (%)	5 (36)

*TAM* = Topical antiglaucoma medications; *AC* = Anterior chamber

## Discussion

When compared, the OVD-free method with patent irrigator introduced in the present study was different from the hydroimplantation technique reported previously. The hydroimplantation technique first introduced in 2010 by Tak [25] uses an irrigation cannula for intraocular lens (IOL) implantation in the aphakic eye after phacoemulsification. Its efficacy and safety has been evaluated in randomized controlled trials for IOL (including toric IOL) implantation [26, 27] and both studies found that this technique is safe and effective with reduced surgical time and cost when compared to the conventional implantation using OVDs. To the best of our knowledge, this OVD-free method introduced in our study is the first reported patented method specific for posterior chamber phakic IOL implantation. The phakic state required better infusion and maintained the anterior chamber since the space of posterior chamber is smaller and extra caution is needed to protect both endothelial cells and clear crystalline lens.

In this study, the clinical application of this OVD-free method provided preliminary data to evaluate its efficacy and safety for ICL implantation in myopic eyes. The clinical outcomes and complications assessment provided the first evidence for a safer method of ICL implantation compared to the standard method. We believe favorable outcomes in this study were a prerequisite for the initiation of the “Prospective, Multicenter, Randomized Clinical Investigation of a Viscoelastic-free Method for Implantable Collamer Lens (ICL) Implantation to Treat High Myopia: A Pilot Study” (NCT03059043), which was designed to fully compare the OVD-free method with the standard method.

The ICL implantation with the standard method has proved to be an effective, safe, and predictable surgery for myopia correction [24, 28–30]. In our study, the LogMAR CDVA at 3 months after surgery was significantly improved with the safety index to be 1.18 in the OVD-free method group and 1.23 in the standard method group. After surgery, the time course of LogMAR CDVA changes between the two groups were different (*p* = 0.047), better LogMAR CDVA was noted in the OVD-free method group at 1 day (− 0.076, 95% CI − 0.134 to − 0.018; *p* = 0.012), 1 week (− 0.071, 95% CI − 0.135 to − 0.007; *p* = 0.03), but not at 3 months (− 0.046, 95% CI − 0.107 to 0.015; *p* = 0.134). This suggests that the OVD-free method was as safe as the standard method with respect to the visual outcomes at 3 months after surgery and may potentially have the advantage of faster recovery of visual acuity at 1 day and 1 week postoperatively. The smooth control of IOP and relatively quiet anterior chamber resulting from the OVD-free method may explain the difference in early recovery of visual acuity between the two groups.

The postoperative rise in IOP has been reported to be the most common complication after ICL implantation [17–19, 28, 30, 31]. Retained OVDs was the major cause of early (within 24 h postoperatively) acute IOP elevation. Almalki et al. [18] investigated the causes of IOP elevation in 58 of 534 eyes (10.8%) that received ICL. They found that retained OVDs (39.7%) accounted for the most IOP elevations that occurred on the first postoperative day. Senthil et al. [19] analyzed the etiologies of IOP elevation in 33 of 638 eyes after ICL implantation. Although coaxial irrigation and aspiration was used to remove the OVDs during the surgery, 5 eyes (15%) had IOP elevations due to retained OVDs on postoperative day 1, and one eye had pupillary block due to a central hole block by the retained OVDs behind the V4c ICL. In this study, IOP elevation was only observed in the standard method group, and fourteen of twenty-one eyes (66.7%) had IOP > 22 mmHg at 2 h after ICL implantation. With timely appropriate management (Table 3), the IOP declined to the normal range at 1 day postoperatively. The cause of the early acute IOP elevation in the standard method group was considered to be the retained OVDs (100%) since normal vault and no sign of pupillary block were observed. The higher incidence of OVD-related IOP elevation compared to the previous studies in the standard method group may due to: 1. The OVDs (1.7% sodium hyaluronate) used in this study had heavier molecular weights or higher viscosity as compared to the previous study (1% sodium hyaluronate in Almalki’s study [18], 2% hydroxyl propyl methyl cellulose in Senthil’s study [19]), and thus tended to elevate the IOP postoperatively [20, 32]; 2. In minimizing the chance of inadvertent PIOL-crystalline lens touch during the surgery, a gentle manner with manual irrigation was used in this study for OVDs removal, which may increase the chance of OVDs being retained when compared to Senthil’s study [19] that used coaxial irrigation and aspiration to remove OVDs. 3. The definition of the IOP elevation was different among studies. In this study, we defined IOP > 22 mmHg at 2 h as IOP elevation. A more restricted definition of IOP elevation was used in previous studies such as IOP ≥24 mmHg^18^ or ≥ 22 mmHg on two separate occasions [19], which could possibly omit some cases with mild elevations or transient IOP peaks.

In the OVD-free method group, none suffered IOP elevation in the early postoperative period. Two hours after surgery, an insignificant increase of IOP to 16.3 ± 3.6 mmHg was noted in the OVD-free method group, this was significantly lower than that (25.7 ± 5.7 mmHg) of the standard method group (− 9.4, 95% CI − 12.1 to − 6.8; *p* < 0.001). The time course of IOP changes (Fig. 2) showed that the mean IOP of the OVD-free method group and standard method group both raised up and reached the peak at 2 h (2.2, 95% CI − 0.19 to 4.5; *p* = 0.094, 10.5, 95% CI 7.8 to 13.2; *p* < 0.001, respectively), then quickly declined at 1 day. The inter-group comparisons revealed that the rise of IOP (difference of IOP from baseline level) was significant higher only at 2 h in the standard group (8.3, 95% CI 5.8 to 10.8; *p* < 0.001), and the difference was not significant at 1 day (0.7, 95% CI − 0.7 to 2.1; *p* = 0.303), 1 week (1.2, 95% CI − 0.3 to 2.8; *p* = 0.122) and 3 months (0.5, 95% CI − 0.9 to 1.9; *p* = 0.498). The changing trend of IOP in the two groups indicated that the early acute IOP elevation can be lowered down effectively by temporary treatment in the standard method group, this, again, supported the idea that retained OVDs was the only cause in the standard method group for acute IOP elevation since other etiologies (such as pupillary block or malignant glaucoma) may require extra management. Meanwhile, this suggested that the OVD-free method used in this study can completely eliminate the OVD-related IOP elevation, and thus avoid the necessity and risk of IOP-lowering management, including antiglaucoma medications, anterior chamber drainage and even surgical intervention such as anterior chamber irrigation. These IOP-lowering management strategies along with a closely observed postoperative period may increase economic burden as well as physical and mental suffering to patients in the standard method group.

In our study, the OVD-free method maintained the anterior chamber by using continuous infusion with BSS through a side port. The efficacy of this method has been demonstrated by the success of the surgeries in all eyes. In the future, the safety of this method will be further investigated. Other than maintaining the stability of the anterior chamber, one of the main aspects of OVD use was the protection of intraocular structures such as corneal endothelial cells in intraocular surgery [20, 33] or clear crystalline lens in ICL implantation. Given the inhibition of in vivo mitosis in humans, the issue regarding the protection of corneal endothelial cells is critical [34]. Whether the OVD-free method can be equally safe compared to the standard method in protecting intraocular structures need to be addressed. In this study, the endothelial cell density in the OVD-free method group fell from 2522 ± 281 cells/mm^2^ preoperatively to 2407 ± 226 cells/mm^2^ 3 months postoperatively (twelve eyes, paired t test, − 115, 95% CI − 295 to 65; *p* = 0.187). Although the difference was not significant, the calculated mean percentage of endothelial cell loss was 4.6% at 3 months. Some discrepancies exist within the literature regarding endothelial cell loss secondary to ICL implantation. Gao et al. [7] reported 2.0% endothelial cell loss at approximately 6 months postoperatively. Alfonso et al. [24] reported 8.5% endothelial cell loss 6 months postoperatively. Lisa et al. [29] found a mean endothelial cell loss of 1.7% at 12 months postoperatively. In the OVD-free method group, a 4.6% endothelial cell loss at 3 months was comparable with the results from previous studies which used the standard method. In addition, in terms of protecting clear crystalline lens without OVDs to avoid cataract formation, the OVD-free method demonstrated good outcomes as there was no cataract formation noted during the 3 month follow up period. Based on the results in this study, a preliminary conclusion can be drawn in that the OVD-free method was safe for ICL implantation without causing additional complications.

Differing from the OVD-free method, the intraoperative use of OVDs for ICL implantation in the standard method increased the overall operation time significantly (*p* < 0.001) because extra time was needed to inject and remove the OVDs. Besides a prolonged surgery, which increased the cost of consumable material and overall risk of intraoperative complications, OVD removal can immediately cause safety issues such as early lens opacification by inadvertent PIOL-crystalline lens touching [22] and possible endothelial cell loss by extra aggressive irrigation steps. However, the intraoperative complications in both groups were not fully assessed due to the retrospective nature of this study. The long-term complications of the OVD-free method could mostly be identical to the standard method because the ICL model (V4c) and position of implantation (posterior chamber) were the same in both groups. The long-term complications after ICL implantation with standard method have been fully evaluated by several studies [29, 30], therefore, the intraoperative complications and short-term complications should be a major concern in the current study so as to investigate efficacy and safety of a newer surgical technique. The current study has demonstrated that all surgical procedures were performed uneventfully, and no pupillary block, cataract formation or pigment dispersion syndrome was seen during the 3-month observation period.

Our study had several limitations. As this was a retrospective study of 49 cases, eyes in both groups were not randomly assigned. Clinical data was incomplete and the sample size was relatively small: postoperative endothelial cell density was only partially available in the OVD-free method group, and the vaults of all the eyes were not completely quantified. We addressed all of these shortcomings in the design of the prospective, multicenter, randomized study (NCT03059043), including randomization and measuring the vaults with anterior segment optical coherence tomography.

## Conclusions

In summary, the results herein indicated that the OVD-free method was safe and efficient for ICL implantation in myopic eyes. It may be a safer method of ICL implantation compared to the standard method by completely eliminating OVD-related complications without causing significant endothelial cell loss and extra cataract formation. Both aspects, in turn, were of benefit for both patients and surgeons alike. However, these results should be confirmed by a prospective, multicenter, randomized study in the future.
